# Ocean Chlorophyll-*a* Concentration and the Extension of the Migration of Franklin’s Gulls (*Leucophaeus pipixcan*) in Southern South America

**DOI:** 10.3390/ani16020301

**Published:** 2026-01-19

**Authors:** María P. Acuña-Ruz, Julian F. Quintero-Galvis, Angélica M. Vukasovic, Jonathan Hodge, Cristián F. Estades

**Affiliations:** 1 Center for Earth and Space, Facultad de Ingeniería y Ciencias, Universidad Adolfo Ibañez, Santiago 7550344, Chile; 2 Departamento de Ciencias, Facultad de Artes Liberales, Universidad Adolfo Ibáñez, Santiago 7550344, Chile; 3 Millennium Nucleus of Patagonian Limit of Life (LiLi), Valdivia 5090000, Chile; 4 Laboratorio de Ecología de Vida Silvestre, Facultad de Ciencias Forestales y Conservación de la Naturaleza, Universidad de Chile, Santiago 8820808, Chile

**Keywords:** waterbirds, facultative migration, estuaries, Chile, Peru

## Abstract

Although many long-distance migratory birds choose stable wintering sites and staging posts, irruptive migrants may exhibit considerable interannual variability in their migratory patterns, often depending on food availability. The Franklin’s gull (*Leucophaeus pipixcan*) is a common long-distance migrant along Chile’s coast during the austral summer. Using census data from three estuaries in central Chile (2006–2023), we analyzed variation in summer populations in relation to chlorophyll-*a* (chl-*a*) concentration along the migration route, used as a proxy for food availability. The best model predicting the number of gulls reaching Chile included a negative effect of chl-*a* concentration on the Peruvian coast (0–10° S) during winter (June–July). Considering the time lag associated with the transformation of phytoplankton into seagull food, this result suggests that primary productivity along the route may influence how far south these birds migrate in search of food. We also found a negative correlation between the summer abundance of Franklin’s gulls in Chile and an eBird index for the species in Peru during the same period, suggesting redistribution of individuals between the two countries in response to resource availability. Models such as ours provide a useful tool for understanding and managing populations of migratory waterbirds.

## Simple Summary

Some migratory birds do not reach the same destination every year. Instead, they may change their itinerary depending on environmental conditions, especially food availability. Franklin’s gull (*Leucophaeus pipixcan*) is among these species, arriving in variable numbers on the coast of Chile during the southern summer. In this study, we used 18 years of bird-count data from three estuaries in central Chile to investigate whether ocean conditions influence the number of gulls reaching the area. We focused on chlorophyll-*a* (chl-*a*), a pigment found in phytoplankton, which is commonly used as an indicator of ocean primary productivity and food availability for marine food webs. We found that in years when chl-*a* concentrations were higher along the Peruvian coast during winter, fewer gulls continued their migration to Chile. Then, when productivity was lower in Peru, more birds arrived in Chile. This suggests that Franklin’s gulls adjust how far they migrate depending on where food is more abundant along their route. These findings provide evidence of how ocean productivity can contribute to bird migration, highlighting the need to consider oceanographic conditions when planning conservation strategies for migratory species in the face of environmental change.

## 1. Introduction

Migration is an important life-history event for many birds [1,2], during which migrants benefit from temporal variation in the profitability of different habitats [3]. Bird migratory behavior is controlled by endogenous (e.g., genetics, “circannual clocks”, etc.) and exogenous (e.g., weather, food availability, etc.) factors [4]. The relative combination of the latter mechanisms results in species being obligate (regular) or facultative (irregular) migrants [4,5,6]. Usually, obligate migrants are associated with predictable food supplies, whereas facultative or irruptive migrants rely on food resources whose abundance varies over time and space from one year to another [2].

Expressions of facultative migration include species/populations that may or may not migrate depending on food availability in their breeding habitats [7,8], species that migrate following moving food sources [9] or populations that migrate different distances among years depending on the abundance of food resources [6,10]. While many long-distance migratory birds choose a very stable set of wintering sites and intermediate staging posts, others may show substantial variability in the locations of their selected sites across seasons [11]. In the latter case, the prevalent driver is usually food availability [6,7,12,13,14,15].

Facultative migration refers to flexible, non-obligatory movements in response to environmental variability, in which individuals or populations adjust their migratory behavior across years in response to ecological conditions [8,16,17]. This strategy has been documented in multiple taxa, including subtropical wading birds such as Wood Storks, which migrate in some years and remain resident in others depending on hydrological conditions [17]. An extreme form of plasticity is irruptive migration, characterized by irregular and often large-scale displacements beyond typical ranges, usually triggered by abrupt changes in food availability or climatic anomalies [18,19]. These flexible patterns challenge traditional models of predictable migration and underscore the need to view migration as a dynamic, context-dependent response to environmental fluctuations.

Chlorophyll-*a* (chl-*a*) concentration in the sea is an important indicator of phytoplankton biomass and primary productivity [20]. Moreover, ocean primary productivity has been shown to positively correlate with the distribution of seabirds and their prey [21,22,23,24,25]. While seabirds do not feed directly on phytoplankton, the abundance of phytoplankton is related to that of higher organisms in the food chain [26,27,28].

Mills et al. [29] observed that the abundance of the planktonic euphausiid *Nyctiphanes australis*, the main food source of the red-billed gull (*Chroicocephalus scopulinus*) during its breeding season in New Zealand, was positively associated with the concentration of chl-*a* in surface waters during the austral winter. Similarly, the presence of foraging Cape gannets (*Morus capensis*) has been positively correlated with areas of high chl-*a* concentration in the Benguela upwelling system [28]. Zavalaga et al. [30] found that the distribution of Peruvian boobies (*Sula variegata*) feeding on the continental shelf off Peru was positively correlated with areas of high chlorophyll-*a* concentration.

Chile has one of the longest coastlines in the world, and several migratory bird species use it every year, making it one of the main migratory routes of the Americas [31,32,33]. Most of the country’s coast is influenced by the Humboldt Current, whose upwelling system is among the most productive marine ecosystems in the world [34,35] and an important wintering and stopover region for migratory birds [36,37,38].

Franklin’s gulls (*Leucophaeus pipixcan*) are among the most common long-distance migrants on the Chilean coasts during the austral summer, with tens of thousands of birds congregating in estuarine wetlands [39]. Although timing is regular, the numbers of individuals arriving at estuaries in Central Chile change dramatically from year to year, and these changes are positively correlated across nearby wetlands [39]. Because of this, these authors suggested that a continental-scale redistribution of birds may cause the interannual variation. In this paper, we hypothesized that interannual variability in the number of Franklin’s gulls in central Chile may be associated with changes in primary productivity along the migration route. Specifically, we predicted that during years of high productivity in the northern portion of the species’ wintering range, fewer birds would arrive in central Chile, reflecting facultative (irruptive) migration.

## 2. Materials and Methods

### 2.1. Study Species

Franklin’s gulls breed in prairie marshes in the North-central United States and Southwestern Canada [40,41,42]. During the boreal fall (Sep–Nov) the species migrates south through Oklahoma and Texas, crossing the Gulf of Mexico and the Isthmus of Tehuantepec. From there they travel southward along the Pacific coast, with most birds wintering on the coast of Peru through South-central Chile [43].

In its breeding range, the species mainly feeds on invertebrates (including earthworms and insects) and seeds [44]. Breeding-season feeding ecology and diet have been described in detail [40]. In the wintering areas, Franklin’s gulls feed on insects and marine invertebrates, such as the sand crab (*Emerita analoga*) [45,46]. Some birds feed in the ocean on small fish, such as the Peruvian anchoveta (*Engraulis ringens*) [47,48], and some have even been recorded following trawlers for discards [34]. Winter foraging by larids on surf-zone mole crabs (*Emerita* spp.) has also been documented along the Peruvian coast [45].

### 2.2. Study Area and Data

**Figure 1:**
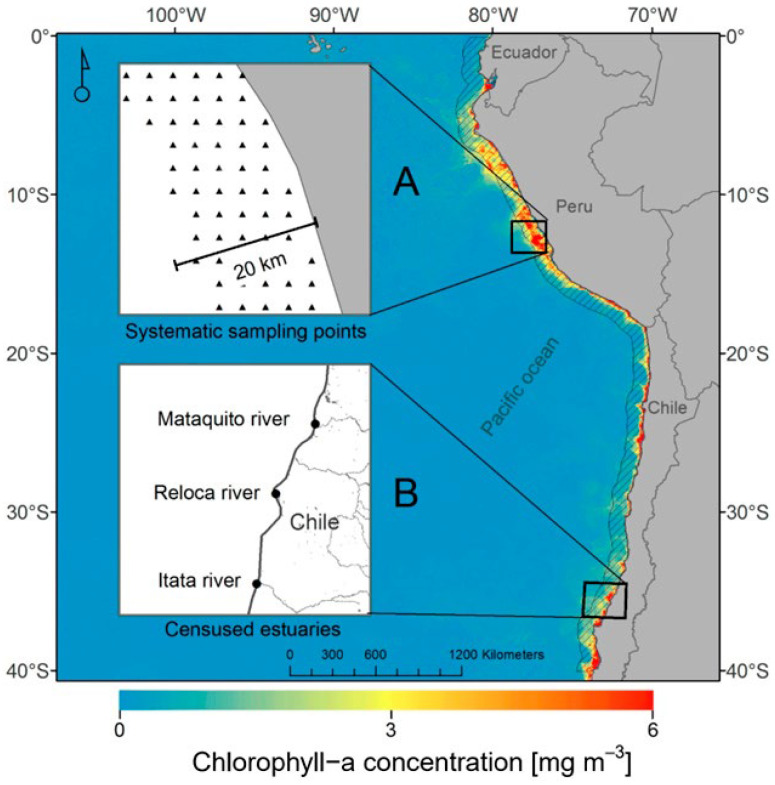
Chlorophyll-*a* (mg m^−3^). (**A**) Systematic sampling points for chl-*a* data in different latitudinal classes from 0° to 40° S and (**B**) estuaries where Franklin’s gull (*L. pipixcan*) census data.

We used Franklin’s gull census data collected in three estuaries in central Chile (Figure 1): the Itata (36°23′ S, 72°51′ W), Mataquito (35°07′ S 72°10′ W) and Reloca (35°43′ S, 72°35′ W) rivers between 2006 and 2023. The region has a Mediterranean climate with rainfall concentrated during the winter season [49]. Average annual temperatures tend to decrease with increasing latitude, from 15 °C to 13 °C [50]. The size of the estuarine area for the Itata, Mataquito and Reloca rivers is 300, 220 and 45 ha, respectively. They all have sand bars and/or are adjacent to beaches where birds roost. The first two estuaries have a marginal cover of marshes and other aquatic vegetation and are surrounded by dunes, meadows, and some agricultural fields. Human presence in these sites is limited to a few artisanal fishermen and some recreational use (i.e., camping and fishing) during the summer season.

Census data were obtained for eight (Reloca and Mataquito) and ten (Itata) campaigns per year, beginning in the austral winter of 2006. At Itata, the study included three campaigns during the summer (December–February), two during the fall migration (April–May), three during the winter (July–August) and two during the spring migration (October–November). Each campaign involved three days of censuses, with two per day (08:00–12:00 h and 14:00–16:00 h). At the remaining sites, one census was conducted in the morning (08:00–12:00 h). During a census, all birds using the estuary, including islets, sandbars, and the immediate shore, were counted using a spotting scope from fixed vantage points [51]. See Estades and Vukasovic [39] for more details on the census procedures.

Although Franklin’s gulls spread out along the coast to feed, large numbers congregate in estuaries to roost and bathe [39], providing a good indicator of the species’ regional abundance. For analysis, we pooled the data from the three estuaries for each date. Finally, for each year, we calculated the average number of gulls recorded during the austral summer (December–February), the period of highest concentration of the species on Chilean coasts.

**Figure 2:**
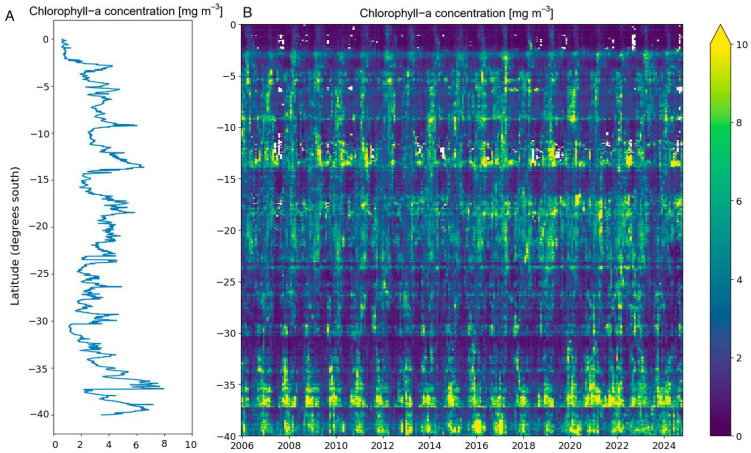
(**A**) Mean chlorophyll along latitude (**B**) Temporal and latitudinal variation of mean Chlorophyll-*a* concentration (mg m^−3^) from 2006 to 2024. South America’s Pacific coast.

Data on ocean chlorophyll-*a* (chl-*a*) were obtained using the Data Cube Chile (https://datacubechile.cl/, accessed on 20 November 2024) which is based on the Open Data Cube (https://opendatacube.org, accessed on 20 November 2024). These tools enabled the extraction of Level 2 processed data from the MODIS/Aqua and Terra Ocean Color sensors (OCI algorithm, [52]) for the period January 2006 to March 2024 (Figure 2). We focused on a 20 km coastal strip along the Pacific coast of South America, dividing it into four latitudinal ranges: 0–10° S, 10–20° S, 20–30° S, 30–40° S. For each band, we calculated bimonthly averages of chl-*a* (mg m^−3^) (e.g., January–February, February–March, March–April). These data were systematically aggregated, yielding a comprehensive dataset representing the mean primary productivity (chl-*a*) along the migratory route of Franklin’s gulls.

The correlation between phytoplankton biomass and that of organisms higher up on the food chain, such as those eaten by seagulls, may show time lags of up to several months [26,53,54]. However, because such time lags were not previously known for our study system, we explored different values ranging from 1 to 12 months. Thus, as predictors, we used 12 bimonthly average values of chl-*a* concentration (i.e., January–February, February–March, …, December–January) for four latitudinal bands. Accordingly, we generated 84 predictor variables (four latitudinal ranges × 12 time periods [months]) evaluated from January 2006 to February 2023.

Additionally, to test whether changes in the number of Franklin’s gulls detected in Chile reflect changes in the species’ global population size, we included an estimate of the abundance of Franklin’s gulls on their breeding grounds during the same season as a covariate. For this purpose, we downloaded the raw Breeding Bird Survey (BBS) data for the species for the years 2006–2023 [55]. From this data we extracted and pooled the counts for all routes in Alberta, Manitoba, Saskatechewan, North Dakota and Utah, regions that harbor the largest breeding populations of the species. Data for 2020 (survey suspended due to the COVID-19 emergency) were estimated by linear interpolation between 2019 and 2021.

Finally, because our hypothesis implies that there might be a redistribution of birds between the coasts of Peru and Chile, we used as a covariate an estimate of the summer abundance of the species in Peru, obtained from the eBird database [56]. eBird is a citizen science program that has been successfully used to study bird migration patterns [57,58]. Thus, from this database (http://ebird.org/content/, accessed on 1 October 2024), we obtained abundance data for December to February, 2006–2023. For each year, we used the average of the maximum weekly records for these three months as an abundance index. Weekly maxima corresponded to the highest single eBird count reported per week across all checklists in Peru. Over the summer studied, the eBird database contained approximately 13 records per week of the species in Peru.

### 2.3. Data Analysis

We used the average abundance of Franklin’s gulls recorded in the three estuaries during the austral summer as the dependent variable. We tested the effect of the 86 (84 + 2) aforementioned predictors on the dependent variable using Generalized Least Squares (GLS) models, accounting for positive temporal autocorrelation (corAR1(form = ~1|year)). For analysis purposes, data on chl-*a* concentration were log-transformed [24]. However, due to severe issues with singularity and non-convergence observed in the data, we opted for Generalized Additive Mixed Models (GAMMs) using the mgcv package (v1.9-4) [59], which are inherently more robust for handling highly complex, non-linear, and over-dispersed count data. The GAM approach also allowed for a flexible, non-linear assessment of continuous predictors. In this paper, we present the results of the GLS and GAMs. All analyses we conducted using the R software version 4.3 [60].

## 3. Results

**Figure 3:**
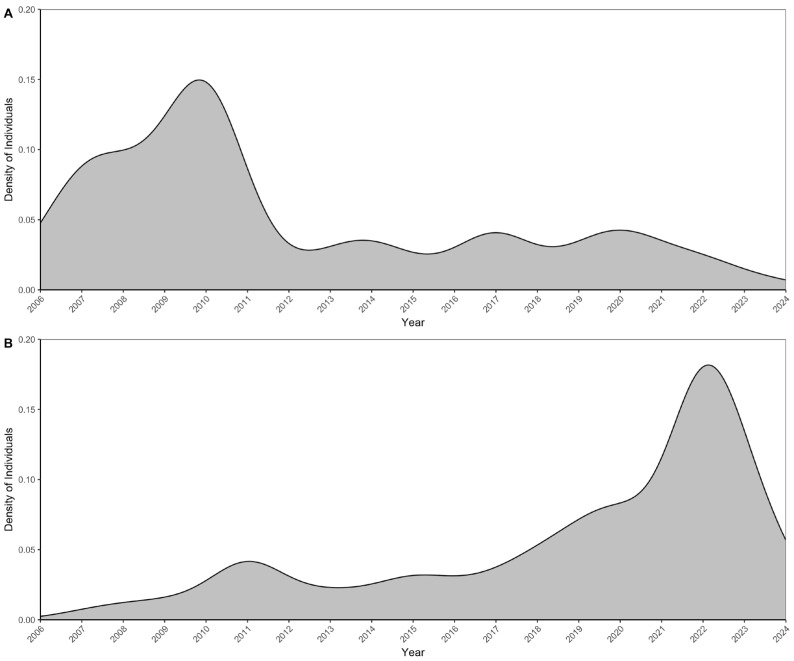
Temporal distribution of individuals (**A**) Franklin’s gulls (*L. pipixcan*) and (**B**) Abundance index for the species in Peru (average of weekly maximum counts during the summer months). The curves represent smoothed density estimates, with each year weighted by its number of individuals (Kernel density estimation weighted by the number of individuals per year). Higher density values indicate periods with greater concentration of individuals.

The abundance of Franklin’s gulls on their wintering grounds in Chile and Peru varied during the study period. The aggregated abundance of the species at estuaries in Central Chile has decreased since the start of sampling, as evidenced in Figure 3A. In contrast, the species abundance index in Peru from 2006 to 2024 shows an increase (Figure 3B).

Due to identified overdispersion and non-normality in the data, we transitioned from a generalized least squares (GLS) model to a generalized additive model (GAM) to provide a more robust analysis. The initial GLS model, which included log-transformed chlorophyll measurements and the covariate Peru, indicated that the coefficients for June–July and July–August were particularly informative, suggesting greater understanding of the underlying patterns in the data (Table S1). Specifically, in the poorly performing model GLS-2 (“AIC” = 67), Chlorophyll during the “June–July” and “July–August” periods showed marginal significance (*p* = 0.0642 and *p* = 0.0610, respectively) (Table S2). This marginal effect, however, was highly unstable and disappeared entirely when the robust non-linear and random effects of the generalized additive model (GAM) were introduced (M1 to M4), underscoring that any subtle seasonal signal is overwhelmed by the inter-annual variability of the system. The best model demonstrated good explanatory power, with an adjusted R^2^-squared of 0.87 and a deviance explained of 87.6% (Table S3). Notably, the coefficients for the months June–July and July–August were statistically significant, with *p*-values of 0.03 and 0.04, respectively, suggesting these variables play a crucial role in influencing the response (Table S4). In contrast, other chlorophyll measures had either non-significant coefficients or less substantial effects. The employed negative binomial distribution effectively addressed the overdispersion observed in the data, affirming the robustness of the model’s estimates and the validity of the identified patterns.

**Figure 4:**
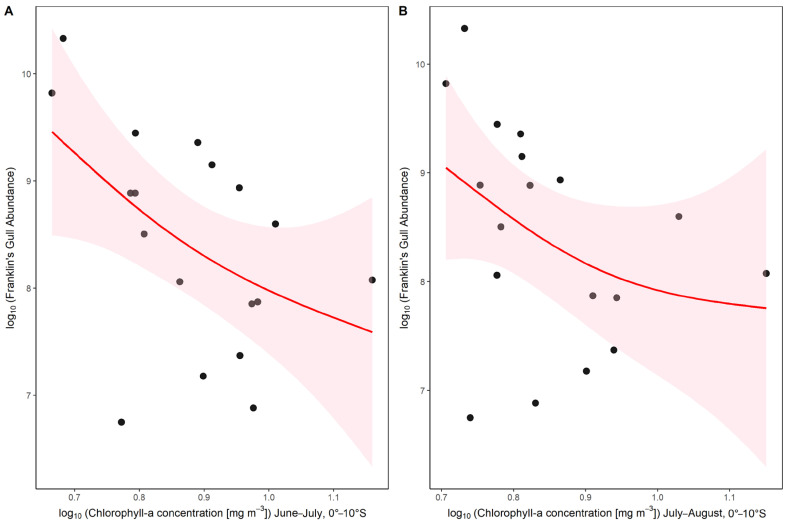
Relationship between log chlorophyll-*a* concentration and Franklin’s Gull abundance for significant period during winter. (**A**) June–July period (0–10° S), and (**B**) July–August period (0–10° S). Black points represent observed data for each year. The red line shows the generalized additive model (GAM) fit, and the shaded area indicates the 95% confidence interval. Both axes are log_10_ transformed. The GAM captures the non-linear relationship between chlorophyll-*a* concentration and gull abundance during both periods.

Among the latter, the best model (lowest AIC) included a negative effect of the logarithm of chl-*a* concentration on the Peruvian coast (0–10° S) during the austral winter (June–July and July–August). Figure 4 shows the relationship between the summer abundance of Franklin’s gulls in central Chile and chl-*a* concentration during winter (June–July and July–August) at latitudes 0–10° S, 2006–2023.

## 4. Discussion

Over almost two decades of monitoring, the numbers of Franklin’s gulls that reach estuaries in central Chile every summer have changed by more than one order of magnitude. As predicted, our analyses showed that a significant portion of this interannual variation can be accounted for by changes in ocean productivity along the migration route. The analysis of the presence data Franklin’s gulls revealed that the temporal dynamics of both migratory bird populations were overwhelmingly governed by annual variability, with local environmental factors, such as Chlorophyll-*a* concentration, showing significant predictive power during winter period.

We used the ocean primary productivity as a (time-lagged) proxy for the abundance of food for Franklin’s gulls. Despite some limitations of such a relationship (e.g., see Grémillet et al. [28]), several studies have shown a positive association between the abundance of different fish species and the ocean-surface concentration of chlorophyll a [22,61,62]. The fact that the latter variable can be efficiently estimated through remote sensing [63,64] makes it an interesting tool for broad-scale and long-term studies such us ours. Importantly, variability in chl-*a* along the Humboldt Current also reflects broader climate–ocean forcing (e.g., changes in upwelling intensity and water-mass structure), providing an explicit link to climatic variability without adding new covariates [35,65].

Our best model indicates a negative effect of winter (June–July and July–August) on the number of gulls arriving in central Chile during the austral summer. Franklin’s gulls reach the coasts of Peru in October-November [39], and the food abundance they encounter is likely associated with primary productivity in the area some time before that. If our interpretation is correct, that would imply a 3–4 month time lag between any moment of a given chl-*a* concentration and the time when that phytoplankton biomass has been transformed into invertebrates (e.g., *Emerita analoga*) or fish (e.g., *Engraulis ringens*) consumed by gulls. Franklin’s gulls rely on marine prey, such as zooplankton and small pelagic fish, during their coastal stay in South America [39]. These resources tend to concentrate in areas of high secondary production following phytoplankton blooms. The timing and strength of this trophic transfer is modulated by upwelling intensity and oxygen-driven vertical habitat compression, which influence prey availability in the Humboldt Current system [65,66]. In this scenario, in years of high winter productivity at the 0–10° S latitude, on their arrival, Franklin’s gulls will find enough food and many of them might decide not to continue their southward migration. If, alternatively, Franklin’s gulls continue to the coast of central Chile due to the abundance of food present in that latitude (30–40° S), during years of food shortage they would have to either return to Peru or continue their migration further south. To the best of our knowledge, neither of the latter has happened during the studied period (authors, pers. observ., communications from different unpublished sources).

Migrant birds may prefer to winter in areas as close to their breeding habitat as possible [6], and the distance these birds will travel is likely governed by physiological mechanisms [10]. Thus, Franklin’s gulls may decide to continue their southward migration to avoid starvation. During the austral summer of 2010–2011, when we recorded the highest numbers of Franklin’s gulls at our study sites, thousands of birds were also observed searching for food in inland wetlands, mine tailings reservoirs and even landfills in different parts of the country (Authors, unpub. data, several personal communications). These observations suggest that a substantial number of birds may have encountered limited food availability along the coast, leading them to explore alternative inland habitats. This behavioral response is consistent with an optimal foraging strategy under conditions of reduced marine prey abundance. It supports our interpretation that high numbers of Franklin’s gulls in central Chile are more likely linked to limited prey availability in Peru rather than to elevated food resources in Chile.

**Figure 5:**
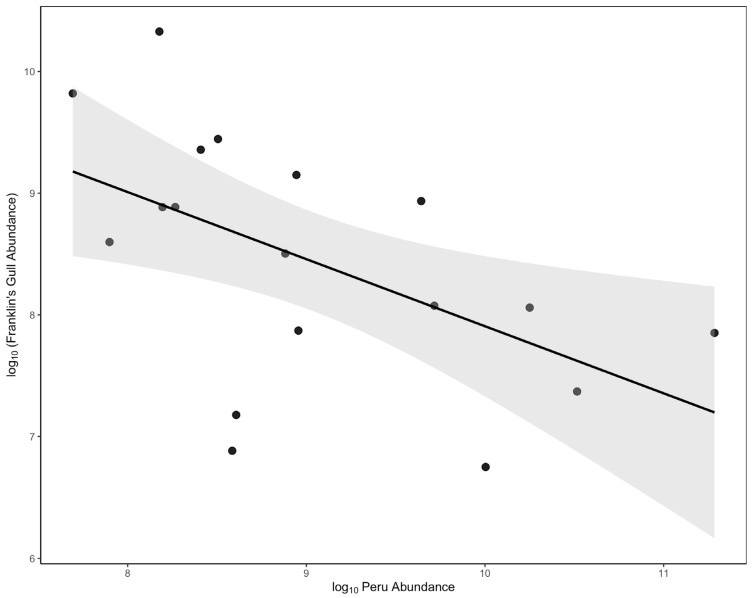
Relationship between Franklin’s Gull abundance in central Chile and an eBird population index for the species in Peru.

The negative correlation between the austral summer abundance of Franklin’s gulls in Peru and Central Chile also support the hypothesis of a redistribution of birds between the coasts of these two countries. As shown in Figure 5, the relationship between species abundance in Chile and the eBird abundance index for Peru during the same period highlights the potential effects of food resource availability on migratory behavior. Although concerns have been expressed in relation to the adequacy of the eBird data for bird population assessments [67], particularly for areas with more sparse datasets, such as Latin America [68], we are confident that the observed relationship reflects a general pattern, due to the large number of years analyzed in this study.

Although it could have been expected that higher numbers of gulls during the boreal summer in North America would translate into more potential migrants heading south and vice versa, the BBS abundance index of Franklin’s gulls in their breeding grounds was not a useful covariate for our model, failing to account for some of the inter annual variation in the numbers recorded in central Chile, a potential explanation for the lack of evidence of such “seasonal interaction” [69], may be because the numbers of individuals of the species that reach the Chilean coasts every year might be a minor proportion of the total amount of Franklin’s gulls that migrate to South America each season.

One characteristic of facultative migration is its apparent unpredictability [2,70,71,72]. However, the evidence presented in this paper shows that a simple model, based on freely accessible data, can help identify the factors underlying the migratory behavior of the Franklin’s gull in Chile. And a better understanding of avian migration patterns can have important implications in the design of effective species conservation measures at different sites along their migration routes [73,74,75].

## 5. Conclusions

Using census data obtained from three estuaries in Central Chile from 2006 to 2023, we showed that the annual variation in the number of Franklin’s gulls observed during the austral winter in this region was negatively correlated with chl-*a* concentrations on the Peruvian coast (0–10° S), which are considered a time-lagged proxy for the abundance of food for the species.

This relationship, in addition to a negative temporal correlation between the winter abundances of Franklin’s gulls in Peru (eBird data) and central Chile (our census data), strongly suggests that during years when the species encounters abundant food resources at its arrival on the Peruvian coast (October–November), few individuals continue their migration further south, and vice versa.

## Data Availability

Part of the data used in this study are publicly available: North American Breeding Bird Survey (https://www.pwrc.usgs.gov/bbs/, accessed on 1 October 2024) and eBird data for Peru (https://ebird.org/region/PE, accessed on 1 October 2024). Census data for estuaries in Central Chile can be obtained from the authors upon request. NASA Goddard Space Flight Center, Ocean Ecology Laboratory, Ocean Biology Processing Group. Moderate-resolution Imaging Spectroradiometer (MODIS) Terra MODIS Level-2 Ocean Color (OC), Version 2022 Data; NASA OB.DAAC, Greenbelt, MD, USA. doi: https://doi.org/10.5067/TERRA/MODIS/L2/OC/2022.0. NASA Goddard Space Flight Center, Ocean Ecology Laboratory, Ocean Biology Processing Group. Moderate-resolution Imaging Spectroradiometer (MODIS) Aqua MODIS Level-2 Ocean Color (OC), Version 2022 Data; NASA OB.DAAC, Greenbelt, MD, USA. doi: https://doi.org/10.5067/AQUA/MODIS/L2/OC/2022.0.

## Supplementary Materials

The following supporting information can be downloaded at: https://www.mdpi.com/article/10.3390/ani16020301/s1, Table S1: Analysis of Generalized Least Squares (GLS) Models for FranklinDEF Presence vs. Chlorophyll-a by Latitude and by Month; Table S2: Coefficient of Generalized Least Squares (GLS) Model GSL 2 of Table S1; Table S3: Comparison of Generalized Additive Model (GAM) Performance and Selection for FranklinDEF; Table S4: Parameter Estimates for the best model of GAM of FranklinDEF. Model M1 Table S3.
