# Supplementary material for: Ocean Chlorophyll-a Concentration and the Extension of the Migration of Franklin’s Gulls (Leucophaeus pipixcan) in Southern South America
Source: Animals (Basel). 2026 Jan 19;16(2):301. doi: 10.3390/ani16020301 (PMC12837980; doi:10.3390/ani16020301)
Supplement: Supplementary file 1 [file animals-16-00301-s001.zip › animals-4061675-supplementary.pdf]

Supplementary Information

María P. Acuña-Ruz [1,*], Julian F. Quintero-Galvis [2,3], Angélica M. Vukasovic [4], Jonathan Hodge [1] and Cristián F. Estades [4,*]

[1] Center for Earth and Space, Facultad de Ingeniería y Ciencias, Universidad Adolfo Ibañez, Santiago 7550344, Chile
[2] Departamento de Ciencias, Facultad de Artes Liberales, Universidad Adolfo Ibáñez, Santiago 7550344, Chile
[3] Millennium Nucleus of Patagonian Limit of Life (LiLi), Valdivia 5090000, Chile
[4] Laboratorio de Ecología de Vida Silvestre, Facultad de Ciencias Forestales y Conservación de la Naturaleza, Universidad de Chile, Santiago 8820808, Chile
* Correspondence: paz.acuna@uai.cl (M.P.A.-R.); cestades@uchile.cl (C.E.); Tel.: +56-992508016 (M.P.A.-R.)

Table S1. Analysis of Generalized Least Squares (GLS) Models for FranklinDEF Presence vs. Chlorophyll-*a* by Latitude and by Month.

**Model ID**	**Formula**	**logLik**	**AIC**	**p-value≈0.06**
Model GLS-1	FranklinDEF~log(Chlor(4 latitude))+PeruDEF max	-25.68	120	None
Modelo GLS-2	FranklinDEF~log(Chlor(4 latitude)+month)+PeruDEFmax	-45.01	67	Chlor_67 (0.0642), Chlor_78 (0.0610)

Table S2. Coefficient of Generalized Least Squares (GLS) Model GSL 2 of Table S1.

**Variable**	**Model (GLS 2) Coefficient**	**SE**	**p-value**
Intercept	30246.78	46026.00	0.5401
log_chlor_1011	154453.78	90920.64	0.1501
log_chlor_1112	-120325.4	68545.47	0.1395
log_chlor_12	23970.24	47988.92	0.6386
log_chlor_23	-142846.3	105078.69	0.2321
log_chlor_34	230721.89	131041.82	0.1386
log_chlor_45	-81406.13	84183.37	0.3780
log_chlor_56	9136.01	68514.10	0.8991
log_chlor_67	-224683.1	94902.92	**0.0642**
log_chlor_78	181336.39	75289.82	**0.0610**
log_chlor_89	77190.44	81270.89	0.3858
log_chlor_910	-128066.06	85260.11	0.1934
PeruDEFmax	-0.01	0.11	0.9519

Note: In bold variable with an interesting coefficient

Table S3. Comparison of Generalized Additive Model (GAM) Performance and Selection for FranklinDEF.

Model ID	Distribution	Formula Structure	R^2 Adjusted	Deviance Explained	AIC	Delta AIC
M1	NegBinomial	log(Chlor_month) + latitude+PeruDEFmax	0.872	87.60%	**342.2**	0
M2	NegBinomial	s(log(Chlor_month))+ latitude+PeruDEFmax	0.865	82.50%	358.36	16.13
M3	Gamma	log(Chlor_month)+ latitude+PeruDEFmax	0.865	82.50%	358.37	16.14
M4	Gaussian	log(Chlor_month)+ latitude+PeruDEFmax	0.831	83.10%	367.2	24.97

Table S4. Parameter Estimates for the best model of GAM of FranklinDEF. Model M1 Table S3.

Variable	M1 (GAM-NB) Coefficient	SE	p-value
Intercept	6.89E+00	5.20E+00	0.2425
log_chlor_1011	1.24E+01	1.03E+01	0.2804
log_chlor_1112	-1.02E+01	7.74E+00	0.2455
log_chlor_12	-1.22E-01	5.42E+00	0.983
log_chlor_23	-1.02E+01	1.19E+01	0.4294
log_chlor_34	2.05E+01	1.48E+01	0.224
log_chlor_45	-3.52E+00	9.51E+00	0.7263
log_chlor_56	-8.90E-03	7.74E+00	0.9991
log_chlor_67	-2.50E+01	1.07E+01	**0.0371**
log_chlor_78	2.21E+01	8.50E+00	**0.0482**
log_chlor_89	3.78E+00	9.18E+00	0.6974
log_chlor_910	-9.03E+00	9.63E+00	0.3912
PeruDEFmax	-9.83E-06	1.28E-05	0.4771

Note: Bold represents values of $p<0.05$
